# p53 switches off pluripotency on differentiation

**DOI:** 10.1186/s13287-017-0498-1

**Published:** 2017-02-28

**Authors:** Tongxiang Lin, Yi Lin

**Affiliations:** 1 Stem Cell Research Center, College of Bee Science, Fujian Agriculture and Forestry University, 15 ShangXiaDian Rd, Fuzhou, Fujian 350002 China; 2 Center for Regenerative and Translational Medicine, The Second Affiliated Hospital (Guangdong Provincial Hospital of Chinese Medicine), Guangzhou University of Chinese Medicine, 111 Dade Rd, Guangzhou, Guangdong 510120 China

**Keywords:** P53, Embryonic stem cells (ESCs), Induced pluripotent stem cells (iPSCs), Cancer stem cells

## Abstract

The role of p53 as “a guardian of the genome” has been well established in somatic cells. However, its role in pluripotent stem cells remains much more elusive. Here, we discuss research progress in understanding the role of p53 in pluripotent stem cells and in pluripotent stem cell-like cancer stem cells. The p53 protein, which plays a key role in embryonic stem cells, was first discovered in 2005. Landmark studies of p53-related reprogramming elucidated this protein’s importance in induced pluripotent stem cells in 2009. The p53-related safety concerns in pluripotent stem cells have been raised in stem cell-based therapy although the use of iPSCs in therapeutic application is promising. Because cancer stem cells have profiles similar to those of pluripotent stem cells, we also describe potential strategies for studies in cancer stem cells and cancer treatments. The new discoveries of p53 family proteins in pluripotent stem cells have made possible stable progress in stem cell transplantation efficiency and safety, as well as treatment strategies targeting cancer stem cells based on pluripotent stem cell technology.

## Highlights

p53 is a switch in embryonic stem cells.

The p53 switch critically inhibits the generation of induced pluripotent stem cells.

Reactivation of p53 is a potential way to treat tumors by inhibiting formation of cancer stem cells.

## Background

Evidence of desire for eternal youthfulness and regeneration has been used in Chinese fairy tales, such as “The Monkey King’s Journey to the West”, for more than 500 years. Although just a fairy tale, the theme of the story can be interpreted to mean “regeneration medicine”, one of the hottest research topics today. The existence of endogenous pluripotent stem cells and embryonic stem cells (ESCs) holds great promise for regenerative medicine. Induced pluripotent stem cells (iPSCs) were first generated from mouse somatic cells in 2006 through viral insertion of four transcription factors: Oct4, Sox2, Klf4, and cMyc [1].

A year later, it was observed that human adult cells were also capable of being converted into pluripotent stem cells under similar conditions [2, 3]. Due to their self-renewal and ability to differentiate into any cell type, human iPSCs have the potential to replace damaged or diseased cells, thus holding great promise for their use in the development of stem cell-based treatments. The ability to design patient-specific human iPSCs minimizes the chance of transplant rejection providing the greatest potential for their use in regenerative medicine. Key findings of iPSC research have changed our views on cell differentiation and provided better models with which to study disease mechanisms, create effective systems for drug screening, enable manufacture of useful cells for transplantation treatments, and provide data for other basic studies and clinical applications. Consequently, the 2012 Nobel Prize in Physiology or Medicine was awarded to Dr. Shinya Yamanaka for the pioneering research on iPSCs and to Sir John Gurdon for his fundamental work on reprogramming cells in frogs [4, 5].

However, despite the great potential of pluripotent stem cell-based cell therapy, some important issues regarding safety concerns have been raised, especially their ability to induce tumorigenesis and teratoma formation [6]. To address the issue of safety and efficiency, much effort has focused on the development of new methods for iPSC generation through the use of integrating and non-integrating recombinant viruses [7–12], DNA expression vectors [13], episomal vectors [14, 15], mini-circle vectors [16], and liposomal magnetofection [17]. Chemical-based methods [18] that result in chemically induced pluripotent stem cells [19] appear to be the most promising methods of reprogramming among the non-DNA methods, which include the use of proteins [20, 21] and mRNA molecules [22]. These advances have significantly improved reprogramming efficiency and, at the same time, directly or indirectly improved iPSC quality. However, these methods do not directly resolve the safety concerns regarding iPSCs, especially tumorigenesis. Furthermore, some studies have shown that the efficiency improvements achieved by disabling the tumor-suppressor p53 largely compromises safety [23].

The protein p53 has been described as “a guardian of the genome” that protects somatic cells from tumorigenesis. However, whether p53 plays the same role in pluripotent stem cells is much more elusive. While efficiency in iPSC generation has been achieved, tumor-related safety studies involving iPSCs are limited. In fact, some iPSC generation methods may even undermine iPSC safety with respect to tumorigenesis. The current use of iPSCs in clinical trials is therefore limited due to tumorigenic risks. We thus discuss the role of p53 in pluripotent stem cells for clinical application and in pluripotent stem cell-like cancer cells, and how pluripotent stem cell technology can be used for cancer treatment.

## p53 is a switch in embryonic stem cells

While the tumor-suppressor p53 activation in somatic cells during stress results in cell-cycle arrest and apoptosis, its activities in pluripotent stem cells remain unexplored. Different roles of p53 in ESCs, however, continue to be discovered. At the end of 2004, p53 was found to play a key role in the differentiation of mouse embryonic stem cells [24], and this has been used to address some of the safety concerns raised regarding the use of iPSCs. DNA damage leads to high activation of p53 expression which suppresses Nanog, a key ESC transcription factor, and induces ESCs to differentiate into cell types that could efficiently execute cell death to remove damaged DNA [24].

When DNA damage occurs in ESCs, phosphorylation of p53 at serine 315 occurs leading to high expression of the p53 protein. In particular, two sequences within the Nanog promoter region are regulated by p53 [24]. Our extensive analysis shows that the activation of p53 inhibits the core transcription factor Nanog, thereby promoting stem cell differentiation in response to DNA damage in ESCs and thus finally inhibiting tumorigenesis.

**Fig. 1:**
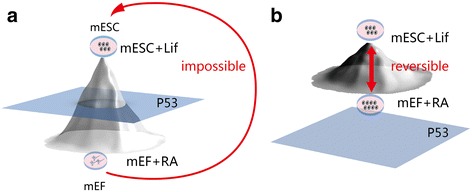
p53 is a switch in embryonic stem cells. Reversible transitions between differentiated cells and embryonic stem cells (*ESCs*) can occur when p53 is switched off. The protein p53 separates differentiated cells from ESCs when the p53 switch is on. **a** Under normal conditions, with an intact p53 signaling pathway, ESCs can differentiate when culture conditions are shifted from mouse ESC (*mESC*) medium containing leukemia inhibitory factor (*Lif*) for mouse ESCs to mouse embryonic fibroblast (*mEF*) medium supplemented with retinoic acid (*RA*). The differentiation process is depicted as cells differentiating from the top to the bottom of the developmental hill. A differentiated cell that has crossed an intact p53 shield cannot return to the top of this hill, which is in the pluripotent stem cell state. **b** Conversely, without p53 (as showed in the figure, the p53 is far from the system and the developmental hill is compressed) differentiated cells can be easily reprogrammed to pluripotent ESCs. For example, differentiated cells can be de-differentiated or reprogrammed to ESCs when the p53 switch is off in humanized p53S315A knock-in mouse ESCs as reported in our previous study [24]

Under normal conditions, with an intact p53 signaling pathway, ESCs can be differentiated by shifting culture conditions from mouse ESC (mESC) medium with mouse leukemia inhibitory factor (Lif) to mouse embryonic fibroblast (mEF) medium supplemented with retinoic acid (RA) (Fig. 1a) [24]. Under normal conditions, it is impossible for a differentiated cell to revert back to a stem cell after it has crossed the intact p53 shield (Fig. 1a).

In contrast, the differentiated cells could be de-differentiated or reprogrammed back to ESC status when the p53 switch is turned off in humanized p53S315A knock-in mouse ESCs (Fig. 1b). This mechanism can be used to allow cells without intact functional p53 to be reprogrammed back to ESCs (Fig. 1b), as shown in the supplemental figures in [24]. These findings were consistent with the first experimental data showing ESC-like induction from mouse testis with p53 loss by Kanatsu-Shinohara and colleagues [25]. Interestingly, although ES-like cells could not be generated from the wild-type germ stem cells after long-term culture, p53 gene knockout cells could produce ES-like cells, which also form teratomas with full pluripotent differentiation potential when transplanted into mice [25].

In contrast to the findings noted above, recent studies have revealed that p53 might be involved in negative feedback pathways in the absence of DNA damage in mouse ESCs. For example, Abdelalim and Tooyama found that p53 was required for mouse ESC self-renewal [26]. In addition, they also determined that the p53 inhibitor pifithrin-alpha might be responsible for the negative feedback and activation of ESC proliferation and self-renewal [26]. These findings show the complexity of p53 function in ESCs.

The detailed mechanism of the pluripotency switch by p53 protein isoforms was elegantly discovered by Ungewitter and Scrable in 2010 [27]. This study showed that the half level expression of the deleted p53 protein version, ¦△40p53, caused a loss of pluripotency in ESCs and initiation of differentiation into somatic cells, while increased dosage of ¦△40p53 maintained pluripotency and inhibited differentiation. The protein activity targeted Nanog and the IGF-1 receptor, which played a central role in the switch between pluripotency and differentiation. In addition to the p53 protein isoform function in the switch, the other two p53 family members, p63 and p73, which were discovered in 1997–1998 [28–31], might also be involved in reprogramming according to recent studies [32, 33].

These two p53 homologues have specific functional differences from p53 and from one another. Mice lacking p63 are born with developmental defects of the limbs and skin caused by impaired ectodermal differentiation during embryogenesis [32], while p73-deficient mice suffer from neurological, pheromonal, and inflammatory defects, but not tumors [33]. A review has discussed the functions of p63 and p73 in the regulation of adult stem-like cells in cooperation with p53 function in cell survival, self-renewal and apoptosis versus senescence, although there is limited information about their complex functions in various aspects of stem cell regulation [34]. It has been observed that p73 is highly expressed in ESCs, suggesting it might also be involved in iPSC generation [35]. On the other hand, p63 has been reported to play important roles in iPSCs through its interactions in several different pathways [36]. Considering the complex interactions among the three family members and related upstream and downstream cell signaling pathways, much more attention should be attached to stem cell safety issues.

## Activation of the p53 switch critically inhibits generation of induced pluripotent stem cells

**Fig. 2:**
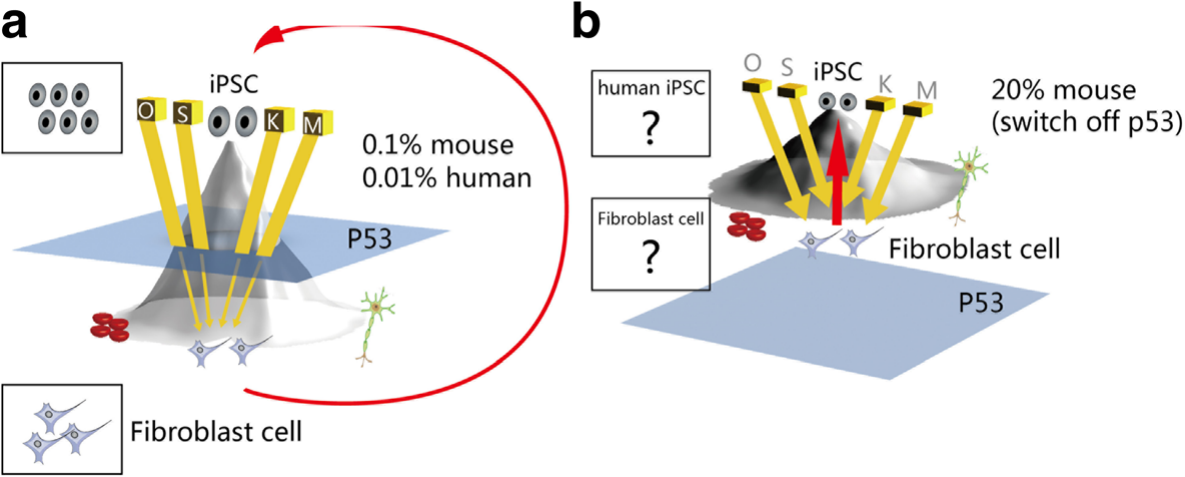
p53 is a critical inhibitor in induced pluripotent stem cells (*iPSCs*). iPSC generation in normal fibroblasts with intact p53 requires the strong effects of four transcription factors, and leaky outcomes are observed. In contrast, iPSC generation coupled with p53 loss requires only two factors and exhibits markedly higher efficiency. **a** During normal development, totipotent stem cells at the top of the developmental hill gradually descend to become somatic cells at the bottom of the hill. A model of human iPSCs is provided in the *upper-left* corner. A model of human fibroblast cells is presented in the *lower-left* corner. Under normal conditions, with an intact p53 signaling pathway, the four transcription factors Oct4 (*O*), Sox2 (*S*), c-Myc (*M*), and Klf (*K*), which are represented by *thick arrows* from the top of the hill, promote the reprogramming of fibroblast cells into iPSCs. However, p53 dramatically attenuates the effects of these factors into much weaker forces, which are depicted as *thin lines*. In particular, iPSC generation across the p53 shield occurs only in certain cases at a low rate of approximately 0.01–0.1%, as reported previously [1–3]. **b** A markedly higher rate of 20% [38] suggests that cell identities shift nearly freely and reversibly between iPSCs and fibroblast cells under conditions of p53 loss. Furthermore, only Oct4 and Sox2 are sufficient for iPSC generation in this situation

Typically, iPSCs are generated through insertion of viral genome into the sequences of a set of four pluripotency-associated genes: Oct4, Sox2, cMyc, and Klf4 [1–3]. Such reprogramming has been successful in mouse and human skin fibroblasts and several other somatic cell types [37], as illustrated in Fig. 2. Human fibroblast cells could be de-differentiated into iPSCs through the overexpression of the four reprogramming factors (Fig. 2a).

However, iPSC generation was initially a very slow and inefficient process, lasting approximately 2 weeks for mouse cells and 4 weeks for human cells. Furthermore, the original conversion of somatic cells into iPSCs occurred at an extremely low rate: approximately 0.01% in human [2, 3] and 0.1% in mouse cells [1]. This low conversion efficiency was also reported in another pioneering work of human iPSC generation [3]. Through overexpression of Oct4, Sox2, c-Myc, and Klf4, human fibroblast cells were de-differentiated into pluripotent stem cells but can dramatically be switched off by overexpressing p53. Therefore, iPSC generation occurs only in leaky incidences in which the cell manages to by-pass the p53 shield (as indicated by the red arrow in Fig. 2a) which supports the finding that, in the absence of p53, the process of iPSC generation would become simple. This has been experimentally proven in research where iPSC generation efficiency was greatly improved in fibroblasts lacking p53, reaching 20% [38], and the parental fibroblast cells assumed iPSC-like status under the ESC culture conditions (Fig. 2b).

p53 expression not only reduces iPSC generation efficiency, but its deletion also replaces the reprogramming factors. It has been reported that deletion of c-Myc from the four-factor gene cocktail could generate iPSCs but that the yield is dramatically low-efficient iPSCs. However, a greatly improved reprogramming efficiency (by 10%) of parental cells generated into iPSCs was seen if p53 was switched off [38]. More research groups have produced iPSCs by using only two factors, Oct4 and Sox2, but under reduced levels of p53 [39]. It was demonstrated that the use of small molecule cell lineage specifiers in their “seesaw model” could be used to reprogram cells without the need for using the four transcription factors [40]. Thus, these data suggest that it is possible to generate iPSCs by turning on pluripotent stem cell genes in a more balanced manner without the need to use the four reprogramming factors [40, 41].

The p53 pathway switches on when DNA damage occurs; hence, only cells having no DNA damage will be able to undergo reprogramming [42, 43]. p53 is therefore so important for the quality control of damage-free iPSCs that its functions should not be ignored, especially in clinical trials [43].

## Reactivation of p53 might control proliferation of cancer stem cells with malignant pluripotency

All iPSC generation proteins (cMyc, Klf4, Nanog, Oct4, and Sox2) are expressed either individually or collectively in many cancer cell types [44, 45]. However, p53 inactivation or deletion significantly increases reprogramming efficiency, as observed in mice derived from iPSCs with p53 knockout [38]. These data, together with many other similar studies, strongly suggest that tumor reprogramming and iPSC generation share similar pathways. Therefore, the risk of tumorigenesis in iPSC-based stem cell application is of major concern. We therefore speculate that cancer stem cells may be generated through a reprogramming.

Accordingly, a recent study found that the production of pluripotent stem cells, which is similar to the creation of the so-called “carcinogenic foci,” can also be used as an experimental model for the study of cancer development and generation of possible treatment strategies [46]. Similar data have also been discussed with iPSC-based cancer stem cells and cancer modeling [47].

Some studies have linked iPSCs and pluripotent stem-like cancer cells. Kitajima et al. observed that deleting retinoblastoma protein (Rb) and N-ras genes in combination with oncogenic Trp53 mutations resulted in cancer stem-like cells, showing elevated expression of embryonic genes, carcinogenic identities, and sensitivity to cancer stem cell target compounds [48]. In many tumor cells, RB and p53 are frequently inactivated. However, deletion of Rb and p53 may result in an undifferentiated state but not in a cancer-like status as found by Kitajima et al. [48].

**Fig. 3:**
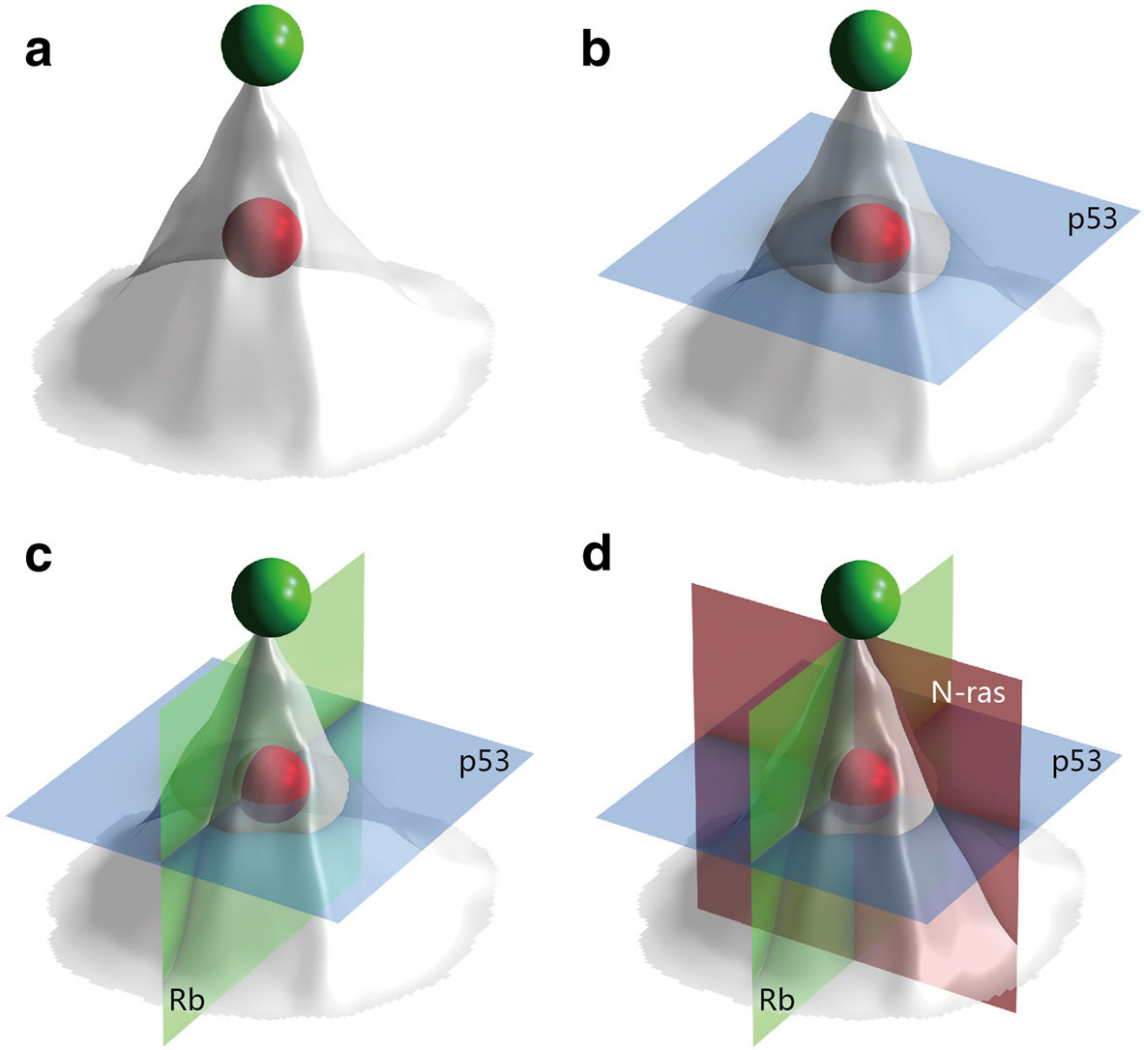
The reactivation of the p53 safety switch rescues cells from a malignant cancer stem cell fate. Research strategies for cancer stem cells might be developed from pluripotent stem cell technology. Based on the recent discovery of cancer stem cells and on iPSC studies, we look forward to the development of treatment strategies for cancer stem cells with pluripotent stem cell-like properties (pluripotent stem cell-like CSCs). The *green sphere* represents normal iPSCs, which are located at the top of the developmental hill. The *red sphere* represents a cancer stem cell with certain safety system defects, such as p53 loss, retinoblastoma protein (*Rb*) deletion, and RAS abnormalities, among others. Pluripotent stem cell-like CSCs may be located at a certain point along the pathway down the developmental hill and may be transferable or may metastasize to other locations. Cancer stem cells might have p53 loss or multiple gene function abnormalities that affect, for example, Rb and N-ras. We hypothesize a method to restrict cancer stem cells with abnormal p53-Rb-Nras signaling pathway disorders. This objective could be achieved through the reactivation of p53, the activation of Rb, and the correction of the abnormal N-ras gene or other genes via a similar process to that used to inhibit cancer stem cells and reach final treatment goals. **a** Tumor stem cells can exhibit abnormal stem cell differentiation (*red sphere*). They can freely transfer to other points on the developmental hill, including the location that represents pluripotent stem cell-like cells. **b** If the normal activity of p53 is restored, p53 can limit cancer stem cells by inducing the loss of most stem cell properties. **c** Other types of cancer stem cells might require the reactivation of two genes, such as Rb and p53, that are restricted or inhibited to cause these cells to lose their metastatic potential. **d** To control the most powerful cancer stem cells, it might be necessary to reactivate all three pathways. These cells will then be forced to completely differentiate, causing them to lose their invasiveness and their metastatic and/or proliferative potential

If these data are representative of tumor or cancer stem cell initiation, then strategies based on pluripotent stem cell technology investigating cancer stem cells might be developed for cancer study and treatment (Fig. 3). In Fig. 3, the green sphere represents a normal iPSC, which is located at the top of the developmental hill. In contrast, the red ball represents a cancer stem cell with certain safety system defects, such as p53 loss, RB deletion, N-RAS abnormalities, and others. The pluripotent stem-like cancer stem cells (CSCs) may be located in an unknown location inside the body and may be transferable or metastasize to other locations (Fig. 3). We therefore suggest the use of methods with the ability to corner cancer stem cells, such as the reactivation of p53, the activation of Rb, and the correction of the abnormal N-ras gene or other genes which may inhibit cancer stem cells and achieve the final goal of the treatment (Fig. 3).

Shown as a red sphere, the tumor stem cell can freely transfer from a point in the developmental hill to some other point, including pluripotent stem-like and metastasis-like status. Once the normal activity of p53 is reactivated, the pluripotent stem-like status can be restricted and the cancer stem cell loses most of its stem cell properties. Previous findings have shown that activation of p53 by nutlin, a small-molecule antagonist of MDM2, leads to the rapid differentiation of human ESCs [49]. Because of the existence of several p53 mutations, other compounds, interfering RNAs, or antibodies specifically targeting the mutant p53 pathways must be developed.

A well-designed paper published a discovery that the undifferentiated state was induced by Rb-p53 double inactivation in mouse somatic cells in 2015 [50]. Using inactivated retinoblastoma tumor suppressor protein (Rb) together with trp53 mutant in vivo and in vitro models, they found that Rb-p53 double inactivation resulted in an undifferentiated reprogramming but without carcinogenic conversion [48]. They built triple abnormality Rb(–/–):N-ras(–/–) MEFs with carcinogenic mutation in Trp53, termed RN6 cells. The RN6 cells showed sphere formation potential, very high expressions of embryonic genes, and appeared to be carcinogenic [48]. Furthermore, RN6 cells were sensitive to specific agents that targeted cancer stem cells [48]. These findings suggest that the genetic interaction between Rb and p53 determines the undifferentiated property, and the triple gene abnormality leads to pluripotent cancer stem cell; thus, the triple genes worked together to make tumorigenic pluripotent cancer stem cell. These data further suggested that these genes might be targets for cancer stem cell-based treatments. Pluripotent-like cancer stem cells showing that malignancy can only be controlled when p53-Rb-Nras pathways are reactivated so that the cells are forced to develop into differentiated cells that are no longer invasive or metastatic, as illustrated in Fig. 3.

## Conclusion: the p53 switch critically impacts pluripotent stem cell applications

The protein p53 has long been established as a key factor for the safety of adult cells. However, recent studies of pluripotent stem cells, including ESCs and iPSCs, have found that p53 is also a key player in pluripotent stem cell differentiation and in reprogramming to generate induced pluripotency. Given what is currently known regarding the generation of stem cells, p53-related activities must be carefully assessed in pluripotent stem cell-based research and applications.

Pluripotent stem cells, including ESCs and iPSCs, are capable of transforming into malignant teratomas, carcinomas, or CSCs once p53 is not controlled. In contrast, the activation of p53 alone or in association with other factors is capable of inhibiting cancer via inhibition of stem cell-related mechanisms. As such, the use of p53 is a potential therapeutic strategy in the management of cancer by maintaining normal and genetically safe cells.

Reactivation of p53 might control and rescue cells from switching into cancer stem cells with malignant pluripotency. However, due to other p53 protein isoforms, such as the deleted p53 isoform mentioned above [27], we should also carefully check which of the p53 isoforms regulate stem cell pluripotency in addition to improving p53 protein stability and activity. Other p53 family members, including p63 and p73, also have opposite functional isoforms which can also interfere with p53 proteins [28–36].

As discussed above, undifferentiated reprogramming resulting from Rb-p53 double inactivation might be a major target of the cancer stem cell treatment strategy. The triple abnormality RN6 cells showed not only pluripotency but also cancer formation potential. RN6 cells with pluripotency and tumorigenesis have already been found to be sensitive to agents that target against cancer stem cells. Targeting the pluripotency as a whole to deal with cancer stem cells, similar to the cell RN6 in which the p53 proteins might be at the center of the regulation target, would be a good strategy. Since p53 switches off pluripotency during differentiation plays major roles in pluripotent stem cells and in pluripotent cancer cells, the activated p53 switch in cancer stem cell management should be applied to cancer treatment.

## Abbreviations

CSC: Cancer stem cell
ESC: Embryonic stem cell
iPSC: Induced pluripotent stem cell
N-ras: Neuroblastoma RAS viral oncogene homolog (the N-ras proto-oncogene is a member of the Ras gene family)
p53: Tumor protein p53
Rb: Retinoblastoma protein
Trp53: Transformation-related protein 53

## References

[1] Takahashi K, Yamanaka S (2006). Induction of pluripotent stem cells from mouse embryonic and adult fibroblast cultures by defined factors. Cell..

[2] Takahashi K, Tanabe K, Ohnuki M, Narita M, Ichisaka T, Tomoda K (2007). Induction of pluripotent stem cells from adult human fibroblasts by defined factors. Cell..

[3] Yu J, Vodyanik MA, Smuga-Otto K, Antosiewicz-Bourget J, Frane JL, Tian S (2007). Induced pluripotent stem cell lines derived from human somatic cells. Science..

[4] Briggs R, King TJ (1952). Transplantation of living nuclei from blastula cells into enucleated frogs’ eggs. Proc Natl Acad Sci U S A..

[5] The Nobel prize in physiology or medicine 2012. http://www.nobelprize.org/nobel_prizes/medicine/laureates/2012/press.html. Accessed 22 Sep 2016.

[6] Simerman AA, Perone MJ, Gimeno ML, Dumesic DA, Chazenbalk GD (2014). A mystery unraveled: nontumorigenic pluripotent stem cells in human adult tissues. Expert Opin Biol Ther..

[7] Fusaki N, Ban H, Nishiyama A, Saeki K, Hasegawa M (2009). Efficient induction of transgene-free human pluripotent stem cells using a vector based on Sendai virus, an RNA virus that does not integrate into the host genome. Proc Jpn Acad Ser B..

[8] Kaji K, Norrby K, Paca A, Mileikovsky M, Mohseni P, Woltjen K (2009). Virus-free induction of pluripotency and subsequent excision of reprogramming factors. Nature..

[9] Loh YH, Yang JC, De Los Angeles A, Guo C, Cherry A, Rossi DJ (2012). Excision of a viral reprogramming cassette by delivery of synthetic Cre mRNA. Curr Protoc Stem Cell Biol.

[10] Stadtfeld M, Nagaya M, Utikal J, Weir G, Hochedlinger K (2008). Induced pluripotent stem cells generated without viral integration. Science..

[11] Woltjen K, Michael IP, Mohseni P, Desai R, Mileikovsky M, Hämäläinen R (2009). piggyBac transposition reprograms fibroblasts to induced pluripotent stem cells. Nature.

[12] Zhou W, Freed CR (2009). Adenoviral gene delivery can reprogram human fibroblasts to induced pluripotent stem cells. Stem Cells..

[13] Okita K, Nakagawa M, Hyenjong H, Ichisaka T, Yamanaka S (2008). Generation of mouse induced pluripotent stem cells without viral vectors. Science..

[14] Okita K, Matsumura Y, Sato Y, Okada A, Morizane A, Okamoto S (2011). A more efficient method to generate integration-free human iPS cells. Nat Methods..

[15] Yu J, Hu K, Smuga-Otto K, Tian S, Stewart R, Slukvin II (2009). Human induced pluripotent stem cells free of vector and transgene sequences. Science..

[16] Jia F, Wilson KD, Sun N, Gupta DM, Huang M, Li Z (2010). A nonviral minicircle vector for deriving human iPS cells. Nat Methods..

[17] Park HY, Noh EH, Chung H-M, Kang M-J, Kim EY, Park SP (2012). Efficient generation of virus-free iPS cells using liposomal magnetofection. PLoS One..

[18] Hou P, Li Y, Zhang X, Liu C, Guan J, Li H (2013). Pluripotent stem cells induced from mouse somatic cells by small-molecule compounds. Science..

[19] Lin T, Wu S (2015). Reprogramming with small molecules instead of exogenous transcription factors. Stem Cells Int..

[20] Kim D, Kim C-H, Moon J-I, Chung Y-G, Chang M-Y, Han B-S (2009). Generation of human induced pluripotent stem cells by direct delivery of reprogramming proteins. Cell Stem Cell..

[21] Zhou H, Wu S, Joo JY, Zhu S, Han DW, Lin T (2009). Generation of induced pluripotent stem cells using recombinant proteins. Cell Stem Cell..

[22] Warren L, Manos PD, Ahfeldt T, Loh Y-H, Li H, Lau F (2010). Highly efficient reprogramming to pluripotency and directed differentiation of human cells with synthetic modified mRNA. Cell Stem Cell..

[23] Krizhanovsky V, Lowe SW (2009). Stem cells: the promises and perils of p53. Nature..

[24] Lin T, Chao C, Saito S, Mazur SJ, Murphy ME, Appella E (2005). p53 induces differentiation of mouse embryonic stem cells by suppressing Nanog expression. Nat Cell Biol..

[25] Kanatsu-Shinohara M, Inoue K, Lee J, Yoshimoto M, Ogonuki N, Miki H (2004). Generation of pluripotent stem cells from neonatal mouse testis. Cell..

[26] Abdelalim EM, Tooyama I (2012). The p53 inhibitor, pifithrin, suppresses self-renewal of embryonic stem cells. Biochem Biophys Res Commun..

[27] Ungewitter E, Scrable H (2010). Delta40p53 controls the switch from pluripotency to differentiation by regulating IGF signaling in ESCs. Genes Dev.

[28] Kaghad M, Bonnet H, Yang A, Creancier L, Biscan J-C, Valent A (1997). Monoallelically expressed gene related to p53 at 1p36, a region frequently deleted in neuroblastoma and other human cancers. Cell..

[29] Schmale H, Bamberger C (1997). A novel protein with strong homology to the tumor suppressor p53. Oncogene..

[30] Trink B, Okami K, Wu L, Sriuranpong V, Jen J, Sidransky D (1998). A new human p53 homologue. Nat Med..

[31] Yang A, Kaghad M, Wang Y, Gillett E, Fleming MD, Dotsch V (1998). p63, a p53 homolog at 3q27-29, encodes multiple products with transactivating, death-inducing, and dominant-negative activities. Mol Cell..

[32] Yang A, Schweitzer R, Sun D, Kaghad M, Walker N, Bronson RT (1999). p63 is essential for regenerative proliferation in limb, craniofacial and epithelial development. Nature..

[33] Yang A, Walker N, Bronson R, Kaghad M, Oosterwegel M, Bonnin J (2000). p73-deficient mice have neurological, pheromonal and inflammatory defects but lack spontaneous tumours. Nature..

[34] Fatt MP, Cancino GI, Miller FD, Kaplan DR (2014). p63 and p73 coordinate p53 function to determine the balance between survival, cell death, and senescence in adult neural precursor cells. Cell Death Differ..

[35] Lin Y, Cheng Z, Yang Z, Zheng J, Lin T (2012). DNp73 improves generation efficiency of human induced pluripotent stem cells. BMC Cell Biol..

[36] Alexandrova EM, Petrenko O, Nemajerova A, Romano RA, Sinha S, Moll UM (2013). DeltaNp63 regulates select routes of reprogramming via multiple mechanisms. Cell Death Differ..

[37] Fukusumi H, Shofuda T, Bamba Y, Yamamoto A, Kanematsu D, Handa Y (2016). Establishment of human neural progenitor cells from human induced pluripotent stem cells with diverse tissue origins. Stem Cells Int..

[38] Hong H, Takahashi K, Ichisaka T, Aoi T, Kanagawa O, Nakagawa M (2009). Suppression of induced pluripotent stem cell generation by the p53-p21 pathway. Nature..

[39] Kawamura T, Suzuki J, Wang YV, Menendez S, Morera LB, Raya A (2009). Linking the p53 tumour suppressor pathway to somatic cell reprogramming. Nature..

[40] Shu J, Wu C, Wu Y, Li Z, Shao S, Zhao W (2013). Induction of pluripotency in mouse somatic cells with lineage specifiers. Cell..

[41] Shu J, Deng H (2013). Lineage specifiers: new players in the induction of pluripotency. Genomics Proteomics Bioinformatics..

[42] Levine AJ, Oren M (2009). The first 30 years of p53: growing ever more complex. Nat Rev Cancer..

[43] Li H, Collado M, Villasante A, Strati K, Ortega S, Cañamero M (2009). The Ink4/Arf locus is a barrier for iPS cell reprogramming. Nature..

[44] Amini S, Fathi F, Mobalegi J, Sofimajidpour H, Ghadimi T (2014). The expressions of stem cell markers: Oct4, Nanog, Sox2, nucleostemin, Bmi, Zfx, Tcl1, Tbx3, Dppa4, and Esrrb in bladder, colon, and prostate cancer, and certain cancer cell lines. Anat Cell Biol..

[45] Schoenhals M, Kassambara A, Vos JD, Hose D, Moreaux J, Klein B (2009). Embryonic stem cell markers expression in cancers. Biochem Biophys Res Commun..

[46] Lee D-F, Su J, Kim HS, Chang B, Papatsenko D, Zhao R (2015). Modeling familial cancer with induced pluripotent stem cells. Cell..

[47] Laplane L, Beke A, Vainchenker W, Solary E (2015). Concise review: induced pluripotent stem cells as new model systems in oncology. Stem Cells..

[48] Kitajima S, Kohno S, Kondoh A, Sasaki N, Nishimoto Y, Li F (2015). Undifferentiated state induced by Rb-p53 double inactivation in mouse thyroid neuroendocrine cells and embryonic fibroblasts. Stem Cells..

[49] Maimets T, Neganova I, Armstrong L, Lako M (2008). Activation of p53 by nutlin leads to rapid differentiation of human embryonic stem cells. Oncogene..

[50] Han S, Woo JK, Jung Y, Jeong D, Kang M, Yoo Y-J (2016). Evodiamine selectively targets cancer stem-like cells through the p53-p21-Rb pathway. Biochem Biophys Res Commun..

